# Disagreement between patient‐ and physician‐reported outcomes on symptomatic adverse events as poor prognosis in patients treated with first‐line cetuximab plus chemotherapy for unresectable metastatic colorectal cancer: Results of Phase II QUACK trial

**DOI:** 10.1002/cam4.3564

**Published:** 2020-11-21

**Authors:** Akira Ooki, Satoshi Morita, Akihito Tsuji, Shigeyoshi Iwamoto, Hiroki Hara, Hiroaki Tanioka, Hironaga Satake, Masato Kataoka, Masahito Kotaka, Yoshinori Kagawa, Masato Nakamura, Tatsushi Shingai, Masashi Ishikawa, Yasuhiro Miyake, Takeshi Suto, Yojiro Hashiguchi, Taichi Yabuno, Masahiko Ando, Junichi Sakamoto, Kensei Yamaguchi

**Affiliations:** ^1^ Department of Gastroenterological Chemotherapy Cancer Institute Hospital of Japanese Foundation for Cancer Research Tokyo Japan; ^2^ Department of Biomedical Statistics and Bioinformatics Kyoto University Kyoto Japan; ^3^ Department of Medical Oncology Kagawa University Kita Japan; ^4^ Department of Surgery Kansai Medical University Kouri Hospital Neyagawa Japan; ^5^ Department of Gastroenterology Saitama Cancer Center Saitama Japan; ^6^ Department of Clinical Oncology Kawasaki Medical School Kurashiki Japan; ^7^ Cancer Treatment Center Kansai Medical University Hospital Osaka Japan; ^8^ Department of Surgery National Hospital Organization Nagoya Medical Center Nagoya Japan; ^9^ Gastrointestinal Cancer Center Sano Hospital Kobe Japan; ^10^ Department of Surgery Kansai Rosai Hospital Amagasaki Japan; ^11^ Comprehensive Cancer Center Aizawa Hospital Matsumoto Japan; ^12^ Department of Surgery Osaka Saiseikai Senri Hospital Suita Japan; ^13^ Department of Surgery Shikoku Central Hospital Shikokuchuo Japan; ^14^ Department of Surgery Osaka Minato Central Hospital Osaka Japan; ^15^ Department of Surgery Yamagata Prefectural Central Hospital Yamagata Japan; ^16^ Department of Surgery Teikyo University School of Medicine Tokyo Japan; ^17^ Department of Surgery Yokohama Municipal Citizen’s Hospital Yokohama Japan; ^18^ Department of Advanced Medicine Nagoya University Hospital Nagoya Japan; ^19^ Tokai Central Hospital Kakamigahara Japan

**Keywords:** cetuximab, chemotherapy, colorectal cancer, patient‐reported outcome

## Abstract

The status and prognostic value of the disagreement between physician and patient assessments of symptomatic adverse events (AEs) remain unclear for patients with metastatic colorectal cancer treated with first‐line cetuximab plus chemotherapy. Paired data on patient‐reported outcomes using the EORTC QLQ‐C30 and physician‐reported outcomes using the NCI‐CTCAE for eight symptomatic AEs (fatigue, pain, insomnia, dyspnea, constipation, appetite loss, nausea/vomiting, and diarrhea) were collected from a prospective trial assessing the relationships between treatment efficacy, AEs, and quality of life. The overall agreement rates between patient and physician reporting at 4 weeks ranged from 40.2% to 76.5% for 129 patients. The level of agreement based on Cohen's κ statistics was slight to poor for dyspnea, pain, fatigue, and insomnia, while it was moderate to fair for the remaining AEs. No clinicopathological characteristics of disagreement were found. The underreporting by physicians ranged from 12.5% (nausea/vomiting) to 56.7% (fatigue). The 2‐year overall survival (OS) rate was more favorable for patients with high agreement than for those with low agreement (71.2% vs. 46.5%, *p* = .016), and the agreement status was an independent factor of OS (HR, 2.31; 95% CI, 1.13–4.71; *p* = .022). For patients who were reported as asymptomatic by the physician, the presence of patient‐reported symptoms resulted in a trend toward poor prognostic outcomes for appetite loss, dyspnea, diarrhea, and constipation. These findings provide the clinical importance of the monitoring of patient‐reported symptoms that can be complementary to physician‐reported data to ensure more accurate clinical outcomes.

## Graphical abstract

The routine use of PROs could be complementary to physician‐reported outcomes to ensure the accurate assessment of symptomatic AEs and facilitate patient‐centered healthcare in clinical practice.

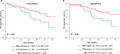

## 1 INTRODUCTION

Colorectal cancer (CRC) has the third highest incidence among cancers and is the second leading cause of cancer‐related deaths.^1^ Approximately 80% to 90% of metastatic CRC patients have unresectable disease,^2^ and anti‐epidermal growth factor receptor antibody (cetuximab or panitumumab) plus chemotherapy is one of the most optimal first‐line treatment regimens, as it has been demonstrated to have significant survival advantages.^3^, ^4^ However, the clinical benefits are limited because mCRC is essentially impossible to cure and because of the development of treatment‐related adverse events (AEs).

The accurate assessment of treatment‐related AEs is crucial in clinical practice for not only an informed evaluation of the treatment based on the balance between the possible risks and expected benefits of treatment but also the appropriate management, including supportive care or treatment modification such as dose reduction or treatment delay. The National Cancer Institute Common Terminology Criteria for Adverse Events (NCI‐CTCAE) is a standard assessment tool of treatment‐related AEs.^5^ However, this assessment is performed by physicians and is not based on direct reports provided by patients themselves; thus, there is a risk of missing the patients’ input even though they are in the best position to comment on their own experiences.^6^ In fact, a growing body of evidence suggests that physicians’ reports of their patients’ AEs may be unreliable and frequently underestimated when compared with the patients’ reports of their own AEs, especially for subjective AEs such as symptoms.^6^, ^7^, ^8^, ^9^, ^10^, ^11^ Consequently, patient‐reported outcomes (PROs) are becoming increasingly relevant to capture a reasonably comprehensive picture of a patient's subjective experience that does not include interpretations of the patient's responses by physicians or anyone else.^12^ Although the substantial variability between physician and patient assessments of symptomatic AEs may depend on specific cancer types and treatment, no prior studies have directly compared patients’ and physicians’ reports of symptomatic AEs in mCRC patients treated with first‐line cetuximab plus chemotherapy. In addition, we and others have previously shown that baseline patient‐reported symptoms are predictors of survival in CRC,^13^, ^14^, ^15^, ^16^ but the prognostic value of the disagreement between patient‐ and physician‐reported outcomes for symptomatic AEs during chemotherapy has not yet been determined.

The QUACK study was prospectively performed to assess the quality of life (QOL) of mCRC patients treated with cetuximab plus chemotherapy in a routine care setting.^13^, ^17^ This study provides the valuable opportunity to assess clinical questions as follows; the aims of this study were (a) to assess the agreement status of symptomatic AEs reported by patients and physicians, (b) to characterize the nature of such disagreements, and (c) to determine whether their disagreement could contribute to poor treatment efficacy and prognostic outcomes. Our findings provide relevant information for patients with mCRC during treatment with cetuximab plus chemotherapy and support that the routine use of PROs may be complementary to physician‐reported outcomes to ensure the accurate assessment of symptomatic AEs and facilitate patient‐centered healthcare in clinical practice.

## 2 PATIENTS AND METHODS

### 2.1 Study design and treatment

The QUACK study is a prospective, multicenter, phase II trial assessing the relationships between treatment efficacy, AEs, and QOL in the first‐line treatment of mCRC with cetuximab plus chemotherapy (FOLFIRI or FOLFOX). Detailed information on the study design has been previously described.^18^ One hundred thirty‐nine patients with mCRC were enrolled from 49 institutions between July 2013 and April 2015, and the primary findings were reported previously.^17^

Chemotherapy regimen (FOLFIRI or FOLFOX) was chosen by the discretion of the physicians in each institution according to their standard clinical practice for treating mCRC. This study was based on the Declaration of Helsinki and the Ethics Guidelines for Clinical Research by the Ministry of Health, Labor, and Welfare in Japan, and written informed consent was obtained from all patients before registration. The study protocol was approved by the institutional review board or ethics committee of each institution.

### 2.2 Symptom assessments

Patient‐reported symptoms were assessed using the European Organization for Research and Treatment of Cancer Quality of Life Questionnaire C30 (EORTC QLQ‐C30) version 3.0, which is a self‐administered, cancer‐specific, and multidimensional questionnaire.^19^, ^20^ The EORTC QLQ‐C30 questionnaire is composed of both multiple and single item scales, including eight symptom scales (fatigue, insomnia, diarrhea, appetite loss, pain, dyspnea, constipation, and nausea/vomiting).^19^ The EORTC QLQ‐C30 symptom items have four response categories (very much, quite a bit, a little, and not at all), and a negative result for a patient‐reported symptom was defined as when patients reported only “not at all” for each of the eight symptom scales. All responses except for “not at all” (i.e., “very much,” “quite a bit,” and “a little”) were categorized as a positive result for patient‐reported symptoms. A linear transformation to standardize the obtained raw data were performed, and the scores ranged from 0 to 100; a higher score indicates a worse level of symptoms.^21^ A change in the scale of at least 10 points was considered clinically meaningful.^22^, ^23^ Simultaneously, physician reporting on each of the corresponding symptomatic AEs was evaluated using the NCI‐CTCAE version 4.0,^5^ and a negative result for a physician‐reported symptom was defined as when physicians reported only “grade 0” for an individual symptom. Four categories were calculated for all eight symptomatic AEs as follows: patient‐positive/physician‐positive, the patients’ reporting was positive using the EORTC QLQ‐C30, and the physicians reported the corresponding symptom as an AE using the NCI‐CTCAE; patient‐positive/physician‐negative, the patients’ reporting was positive, but the physicians did not report the symptom as an AE; patient‐negative/physician‐positive, the patients’ reporting was negative, but the physicians’ reporting was positive; or patient‐negative/physician‐negative, the both reports from patients and physicians were negative. The Dermatology Life Quality Index (DLQI) was used to assess skin‐related QOL.^24^ A change in the DLQI score of at least four points was considered clinically relevant.^25^

QOL assessments were performed at baseline and after 2, 4, 8, 16, and 24 weeks, and patients completed the QLQ assessment before therapy. The survey sheets, including the safety, efficacy, and compliance with treatment, were collected at registration and after 4, 8, 16, and 24 weeks.

### 2.3 Treatment efficacy

The treatment efficacy was evaluated using the computed tomography every 8 weeks during the treatment period. The treatment response was assessed by the investigator using the Response Evaluation Criteria in Solid Tumors (RECIST) version 1.1. Overall response rate (ORR) was defined as the proportion of patients with a partial response (PR) or a complete response (CR) according to the RECIST criteria. Progression‐free survival (PFS) and OS referred to the time from registration to the time of tumor progression and death, respectively. Time to treatment failure (TTF) was defined as the time from registration to the time of treatment discontinuation for any reason, including disease progression, treatment toxicity, patient preference, or death. Post‐progression survival (PPS) was defined as the survival time following progressive disease during first‐line treatment and was obtained by subtracting PFS from OS.^26^

### 2.4 Statistical analysis

Agreement was defined as identical paired responses between the EORTC QLQ‐C30 (a symptom was negative for “not at all” vs. positive for “a little,” “quite a bit,” or “very much”) and NCI‐CTCAE (a symptom was negative for grade 0 vs. positive for grade ≥1 reported by physicians) assessment instruments for each symptomatic AE. Cohen's κ statistic was used to evaluate the status of agreement between the patients’ reporting and physicians’ reporting on the eight symptomatic AEs. The levels of agreement based on Cohen's κ values were classified as almost perfect (0.81 to 1.00), substantial (0.61 to 0.80), moderate (0.41 to 0.60), fair (0.21 to 0.40), slight (0.00 to 0.20), or poor (less than 0.00).^27^ To avoid the Cohen's κ paradox phenomenon, the agreement rate was also calculated in terms of the following: positive agreement, the proportion of cases in which both patients and physicians reported symptom; negative agreement, the proportion of cases in which both reported no symptom; the overall agreement, the proportion of positive or negative agreement.^28^ The status of agreement between the pairs of patient and physician reporting for the eight symptomatic AEs were dichotomized as follows: the overall agreement rates ≥50% and <50% were defined as high and low agreement, respectively.

The Kaplan–Meier method was applied to estimate the prognostic outcomes, and the log‐rank test was used to test the null hypothesis of no difference in outcomes between the populations. The Cox proportional hazard test was performed to assess the effect of specified factors on prognostic outcomes, and the adjusted hazard ratios (HR) and the 95% confidence intervals (CI) were calculated. The continuous data variables are presented as the mean ±the standard error of the mean (SEM), and they were compared using a two‐tailed Student's *t*‐test. Fisher's exact test was used for categorical variables. All statistical analyses were conducted using the JMP 14 software package (SAS Institute).

## 3 RESULTS

### 3.1 Status of agreement between patient and physician reporting on symptomatic AEs

This study was conducted using a secondary data set collected from the prospective QUACK study that assessed the QOL of mCRC patients undergoing cetuximab plus chemotherapy. There were 137 eligible individuals recruited into the QUACK study, and their baseline clinicopathological characteristics have been published.^17^ The median age was 66 years, 95 (69%) were male, and 110 (80%) had an Eastern Cooperative Oncology Group Performance Status (ECOG PS) of 0. Physicians reported almost all patients as grade 0 at baseline according to the NCI‐CTCAE for eight symptoms (fatigue, pain, insomnia, dyspnea, constipation, appetite loss, nausea/vomiting, and diarrhea). Moreover, 55 patients (40%) reported “very much” or “quite a bit” on at least one of the corresponding symptom items of the EORTC QLQ‐C30.^13^ High compliance rates for the EORTC QLQ‐C30 questionnaire were maintained throughout the study period (i.e., 97.9% at baseline, 97.0% at 4 weeks, and 81.1% at 24 weeks).

**FIGURE 1:**
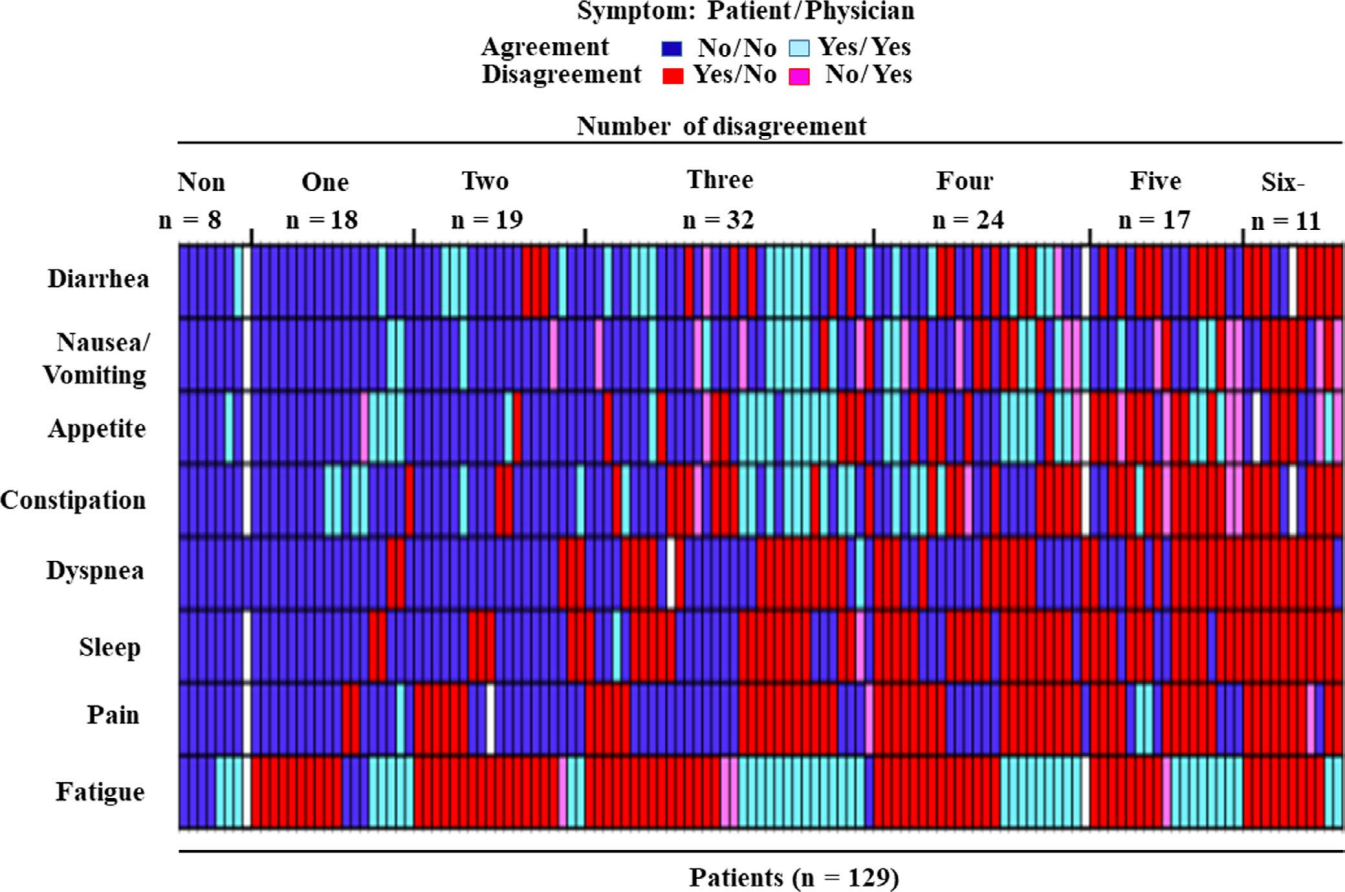
Status of agreement between patient and physician reporting on eight symptomatic AEs (fatigue, pain, insomnia, dyspnea, constipation, appetite loss, nausea/vomiting, and diarrhea). For the patient‐reported symptoms using the EORTC QLQ‐C30, a symptom was defined as negative when patients reported only “not at all,” and the remaining responses (i.e., “very much”, “quite a bit”, and “a little”) were defined as a positive result. For the physician‐reported symptoms using the NCI‐CTCAE version 4.0, a symptom was defined as negative when physicians reported only “grade 0” for an individual symptom, and the other responses were considered a positive result (i.e., grade >0). Four categories were calculated for all eight symptomatic AEs as follows: patient‐positive/physician‐positive (blue), patient‐positive/physician‐negative (red), patient‐negative/physician‐positive (light red), or patient‐negative/physician‐negative (light blue)

At the 4‐week time point after the first administration of cetuximab plus chemotherapy, fatigue was the most frequent AE on which there was disagreement between patient and physician reporting on the eight symptomatic AEs (Figure 1). The overall agreement rates ranged from 40.2% to 76.5% (Table 1). High negative agreement rates (i.e., proportion of cases when no adverse symptom was reported by both patients and physicians), except for fatigue, were observed, whereas positive agreement rates (i.e., proportion of cases when an adverse symptom was reported by both patients and physicians) were low for all symptomatic AEs. Indeed, the negative and positive rates were 48.8% and 2.4% for pain, 46.9% and 0.8% for insomnia, and 57.5% and 0.8% for dyspnea, respectively. Agreement based on Cohen's κ statistics was moderate for appetite loss, diarrhea, and nausea/vomiting (values between 0.41 and 0.60), fair for constipation (values between 0.21 and 0.40), slight for dyspnea, pain, and fatigue (values between 0.00 and 0.20), and poor for insomnia (Cohen's κ value less than 0.00). Similar findings were observed throughout the study period (Table S1). Changes in the scores of more than 10 points and 4 points for the EORTC QLQ‐C30 and skin‐related QOL questionnaires DLQI, respectively, are considered clinically meaningful.^22^, ^23^, ^25^ Then, physician reporting was compared with the clinically relevant change from baseline for each symptomatic AE and skin‐related toxicity. Relatively low agreement was also observed for these AEs (Table 1).

**TABLE 1:** Correlation between patient‐ and physician‐reported outcomes for each symptom at 4 weeks

Symptoms^a^	No. (%) of reports based on the questionnaire^b^				Overall Agreement	Cohen's κ (95% CI)
	Agreement		Disagreement			
	Patient No / Physician No	Patient Yes / Physician Yes	Patient Yes / Physician No	Patient No / Physician Yes		
Fatigue	8 (6.3)	43 (33.9)	72 (56.7)	4 (3.2)	40.2%	0.01 (−0.07–0.09)
Pain	62 (48.8)	3 (2.4)	60 (47.2)	2 (1.6)	51.2%	0.02 (−0.05–0.09)
Sleep	60 (46.9)	1 (0.8)	66 (51.6)	1 (0.8)	47.7%	−0.001 (−0.04–0.04)
Dyspnea	73 (57.5)	1 (0.8)	53 (41.7)	0 (0)	58.3%	0.02 (−0.02–0.06)
Constipation	60 (47.6)	21 (16.7)	40 (31.8)	5 (4.0)	64.3%	0.27 (0.13–0.41)
Appetite	63 (50.0)	29 (23.0)	25 (19.8)	9 (7.1)	73.0%	0.43 (0.27–0.58)
Nausea/Vomiting	78 (60.9)	20 (15.6)	16 (12.5)	14 (10.9)	76.5%	0.41 (0.24–0.59)
Diarrhea	72 (57.1)	21 (16.7)	31 (24.6)	2 (1.6)	73.8%	0.41 (0.26–0.56)

Symptoms	No. (%) of reports based on the difference of scales^c^				Overall Agreement	Cohen's κ (95% CI)
	Agreement		Disagreement			
	Patient No / Physician No	Patient Yes / Physician Yes	Patient Yes / Physician No	Patient No / Physician Yes		
Fatigue	43 (34.1)	21 (16.7)	37 (29.4)	25 (19.8)	50.8%	−0.006 (−0.18–0.17)
Pain	81 (63.8)	0 (0)	41 (32.3)	5 (3.9)	63.8%	0.03(−0.12–0.18)
Sleep	93 (73.2)	1 (0.8)	33 (26.0)	0 (0)	74.0%	0.04 (−0.04–0.12)
Dyspnea	96 (76.2)	0 (0)	30 (23.8)	0 (0)	76.2%	0.0 (NE)
Constipation	80 (63.5)	12 (9.5)	20 (15.9)	14 (11.1)	73.0%	0.241 (0.05–0.43)
Appetite	75 (60.0)	16 (12.8)	12 (9.6)	22 (17.6)	72.8%	0.31 (0.13–0.49)
Nausea/Vomiting	82 (64.6)	17 (13.4)	12 (9.5)	16 (12.6)	78.0%	0.40 (0.22–0.59)
Diarrhea	89 (70.6)	14 (11.1)	14 (11.1)	9 (7.1)	81.7%	0.44 (0.24–0.63)
Skin‐related toxicity	12 (10.4)	33 (28.7)	2 (1.7)	68 (59.1)	39.1%	0.06 (−0.01–0.14)

For skin‐related toxicity, the Dermatology Life Quality Index (DLQI) was used, which was defined as symptomatic if a change in DLQI score of at least four points.

Cohen's κ values: less than 0.00, poor agreement (gray); values between .00 and .20, slight agreement (gray); values between .21 and .40, fair agreement; values between .41 and .60, moderate agreement (bold).

Abbreviation: NE, not evaluable.

^a^ Physician‐reported outcome for each symptom was defined as positive if they had more than grade 1 according to the NCI‐CTC version 4.0.

^b^ Patient‐reported outcome for each symptom was defined as symptomatic if they answered “a little,” “quite a bit,” or “very much” to at least one of the symptom questions and asymptomatic if they answered “not at all” to all of the symptoms according to the EORTC QLQ‐C30.

^c^ Patient‐reported outcome for each symptom was defined as symptomatic if they had a difference of more than 10 points in change scores from baseline and asymptomatic if they had a difference of less than 10 points according to the EORTC QLQ‐C30.

The clinicopathological characteristics in relation to the agreement between patient and physician reporting of symptomatic AEs were examined (Table S2). No tumor‐related, patient‐related, or treatment‐related variables were observed to be significantly different between agreement and disagreement cases.

### 3.2 Physicians’ underestimation of symptomatic AEs

**FIGURE 2:**
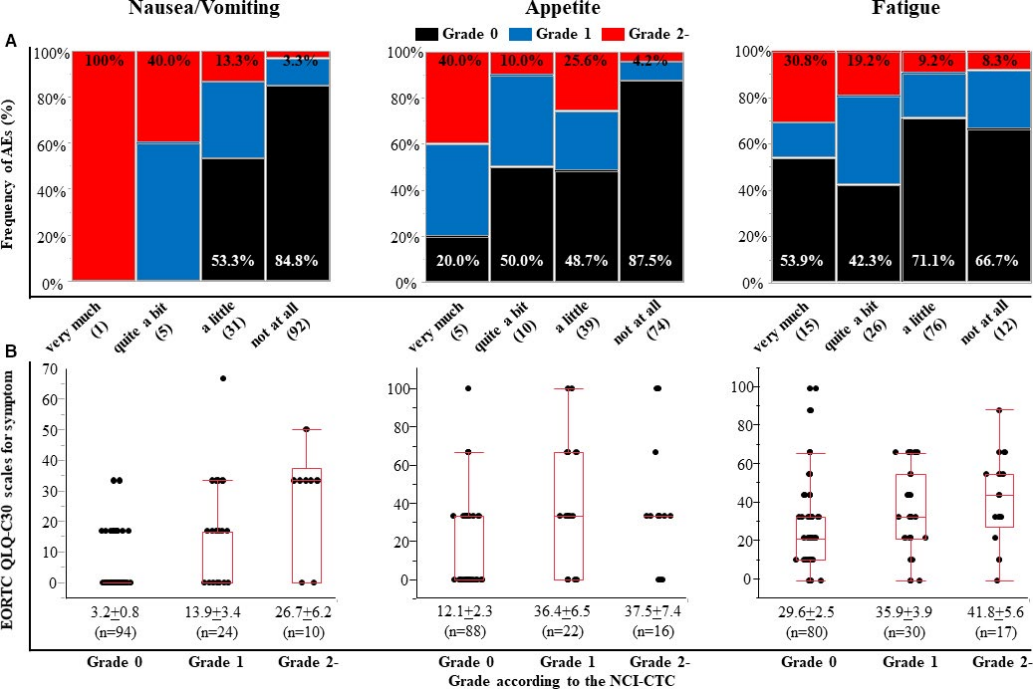
Status of toxic severity between patient and physician reporting on symptomatic AEs. A, Distribution of NCI‐CTCAE grading reported by physicians according to four response categories (very much, quite a bit, a little, and not at all) reported by patients using the EORTC QLQ‐C30 for individual AEs (nausea/vomiting, appetite loss, or fatigue). Black, grade 0; blue, grade 1; red, grade 2. The numbers of patients shown in the parentheses. B, The symptomatic scores of EORTC QLQ‐C30 according to the NCI‐CTCAE grading for individual AEs (nausea/vomiting, appetite loss, or fatigue). Each data point represents the mean ±standard error of the mean.

The reporting incidence was higher for patients than for physicians for all symptomatic AEs, indicating that there was frequent underreporting by physicians (Table 1 and Table S1). Of note, fatigue, pain, dyspnea, and insomnia were frequently reported by patients, but were rarely recognized by physicians throughout the study period; at the 4‐week time point after the first chemotherapy cycle, physician reports (any grade) and patient reports (any severity) of fatigue were documented in 115 (90.6%) and 47 (37.1%) of 127 patients, pain in 63 (49.6%) and 5 (4.0%) of 127 patients, insomnia in 67 (52.4%) and 2 (1.6%) of 128 patients, and dyspnea in 54 (42.5%) and 1 (0.8%) of 127 patients, respectively. Fatigue (56.7%) was the most frequent symptom underestimated by physicians. There was physician underestimation of toxicity even in symptomatic AEs, with faint to moderate agreement based on Cohen's κ statistics; the proportion of underreporting was 12.5% for nausea/vomiting, 19.8% for appetite loss, 24.6% for diarrhea, and 31.8% for constipation. When only patients with any symptoms were assessed, the proportion of physician underestimation was much too high: 44.4% for nausea/vomiting, 46.3% for appetite loss, 59.6% for diarrhea, and 65.6% for constipation. As expected, the proportion of underreporting was considerably high for the symptomatic AEs with slight to poor agreement based on Cohen's κ statistics: 62.6% for fatigue, 95.2% for pain, 98.5% for insomnia, and 98.1% for dyspnea. In addition, physicians often judged the AEs as grade 0 even for symptoms rated as “very much” or “quite a bit” by patients on the EORTC QLQ‐C30 symptom questions (Figure 2A and Figure S1). For patients who reported “quite a bit” or “very much” for each symptomatic AE,

the proportion of physician reporting of an AE as grade 0 was 100% for insomnia and dyspnea, 93.8% for pain, 55.0% for constipation, 46.2% for fatigue, 40.0% for appetite loss, 33.3% for diarrhea, and 0% for nausea/vomiting.

The association of physician‐reported grading using the NCI‐CTCAE instrument with the EORTC QLQ‐C30 scores reported by patients was assessed for each symptomatic AE (Figure 2B and Figure S2). The AEs given a more severe grade by physicians were generally given higher symptomatic scores by the patients, and the differences in the scores between grade 0 and grade 2 were clinically relevant (i.e., differences of more than 10 points).

### 3.3 Association of disagreement status with prognosis

**FIGURE 3:**
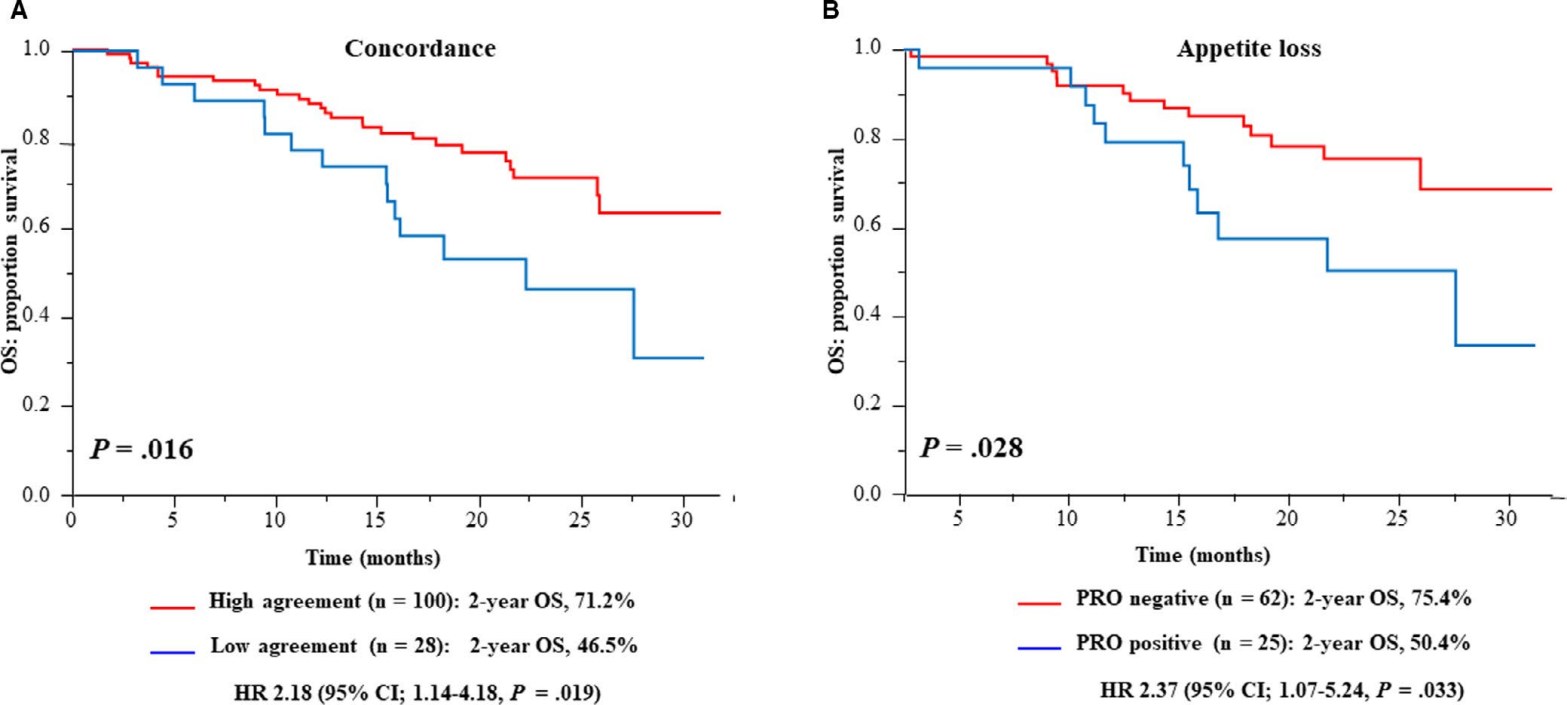
The Kaplan–Meier curves of OS according to the status of A, agreement between patient and physician reporting on eight symptomatic AEs (high vs. low agreement) and of B, PRO for appetite loss in patients who were reported as asymptomatic by the physician (PRO negative vs. positive). Low agreement, defined as discrepant AEs in more than one‐half of the eight symptomatic items

The absolute number of disagreement symptoms between patient and physician reporting was calculated. One or more disagreement was observed in 121 (93.8%) of 129 patients at 4 weeks, and 28 patients had low agreement, defined as discrepant AEs in more than one‐half of the eight symptomatic items (Figure 1). To determine whether disagreement status may provide unique prognostic information at the 4‐week time point after the first administration of cetuximab plus chemotherapy, prognostic analyses were performed between patients with low and high agreement. The data cutoff date was 20 April 2016, and the median follow‐up time was 18.0 months (95% CI, 16.5–19.6). By the cutoff date, 45 and 105 events were observed in relation to OS and PFS, respectively. The 2‐year OS rate was significantly favorable in patients with high agreement compared with in those with low agreement (71.2% vs. 46.5%, HR 2.18, 95% CI, 1.14–4.18, *p* = .016) (Figure 3A). In the multivariable Cox proportional hazard model adjusted for variables (patient's age, tumor differentiation, second‐line chemotherapy, and ECOG PS) that were significant in the univariate analyses, the agreement status remained an independent prognostic factor of OS (HR, 2.31; 95% CI, 1.13–4.71; *p* = .022) (Table 2).

**TABLE 2:** Univariate and multivariable prognostic analyses using the Cox proportional hazard model

Variables	Univariate			Multivariable		
	HR	(95% CI)	*p* value*	HR	(95% CI)	*p* value*
Agreement status^a^						
Low vs. High	2.18	(1.14 to 4.18)	**0.019**	2.31	(1.13 to 4.71)	**0.022**
Differentiation						
poor vs. well/mode	3.23	(1.35 to 7.72)	**0.008**	3.56	(1.32 to 9.61)	**0.012**
Age						
Age ≥70 vs. <70 (years)	2.32	(1.28 to 4.20)	**0.006**	2.55	(1.29 to 5.05)	**0**.007
Second line chemotherapy						
Presence vs. absence	0.51	(0.27 to 0.98)	**0.042**	0.51	(0.25 to 1.07)	**0**.076
ECOG PS						
PS ≥1 vs. PS 0	2.30	(1.21 to 4.38)	**0.011**	1.10	(0.50 to 2.44)	**0**.812
Gender						
Male vs. female	1.24	(0.64 to 2.41)	0.523	**—**	**—**	**—**
Chemotherapy backbone						
mFOLFOX6 vs. FOLFIRI	1.44	(0.75 to 2.75)	0.274	**—**	**—**	**—**
Site of primary tumor						
Colon vs. rectum	1.47	(0.74 to 2.94)	0.274	**‐**	**‐**	**‐**
Primary tumor						
Presence vs. absence	1.32	(0.72 to 2.43)	0.365	**—**	**—**	**—**
CEA						
CEA ≥5 vs. <5	1.37	(0.64 to 3.11)	0.452	**—**	**—**	**—**
Metastatic sites						
Liver only vs. the other	0.61	(0.33 to 1.11)	0.105	**—**	**—**	**—**

Abbreviations: ECOG PS, Eastern Cooperative Oncology Group Performance Status.

^a^ The status of agreement between the pairs of patient and physician reporting for the eight symptomatic AEs were dichotomized as follows: the overall agreement rates ≥50% and <50% were defined as high and low agreement, respectively.

* Cox proportional hazard model. Bold values indicate statistical significance (*p* < .05).

Although no difference was observed in PFS, TTF, or the ORR between the two patient populations, patients with high agreement had a more favorable PPS than those with low agreement (Figure S3 and Table S3). In addition, high agreement at 2 or 8 weeks also showed a trend toward favorable OS and PPS (Figure S4). Age and ECOG PS were significantly different between patients with low and high agreement at 2 weeks, but no clinicopathological characteristics were consistently different throughout the study period (Table S4).

Patients with low agreement had more symptoms because the low agreement was due to frequent physician underestimation. Next, prognostic analyses were performed to evaluate whether many symptoms reflect an unfavorable OS of patients with low agreement. A high PRO was defined as patient reporting symptoms in more than one‐half of the eight symptomatic items. Patients with a high PRO had a poorer OS than those with a low PRO, although the difference was not statistically significant (Figure S5). Of note, low agreement showed a trend toward worse OS despite the status of low and high PRO, supporting that the agreement status is a crucial prognostic factor.

Finally, for patients who were reported as asymptomatic by physicians for an individual symptomatic AE, the association between the presence of patient‐reported symptoms and prognosis was analyzed. Disagreement for appetite loss was associated with worse outcomes (*p* = .028), and the 2‐year OS rate was 75.4% for patients with agreement and 50.4% for patients with disagreement, respectively (HR 2.37, 95% CI, 1.07–5.24), despite having similar PFS and ORR (Figure 3B and Figure S6, and Table S3). Patients with agreement had a more favorable PPS than those with disagreement, although the difference was not statistically significant. Similar findings were also observed for constipation, diarrhea, and dyspnea (Figure S7).

## 4 DISCUSSION

The focus of physicians is generally on the illness and its management, whereas patients with cancer mostly think about the effects of their illness on their lives.^29^ Recently, particular attention is being paid to patient‐centered care, defined as “providing care that is respectful of and responsive to individual patient preferences, needs, and values and ensuring that patient values guide all clinical decisions”.^30^ Although the patient's subjective experience is essential to understand their perspective regarding specific treatments and cancer types, no prior studies have directly compared patient and physician reports of symptomatic AEs during treatment with cetuximab plus chemotherapy even though it is one of the most optimal first‐line treatment regimens for mCRC patients. A better understanding of the clinical impact of disagreement between patient and physician on symptomatic AEs may pave the way for a more patient‐centered approach.

Since the main goal of treatment for mCRC patients is generally palliative, an appropriate treatment of symptoms should be considered. The accurate assessment of AEs can improve clinical management regarding safety, tolerability, and dose modification for patients with mCRC during treatment with cetuximab plus chemotherapy. PROs can provide a different viewpoint and are an essential source of information for treatment toxicity assessments.^31^ In line with previous studies on different cancers and treatments,^8^, ^9^, ^10^, ^11^ in this study, there was high disagreement between patients and physicians for all symptomatic AEs throughout the study period. Although the AEs given more severe grades by physicians were generally also given higher symptomatic scores by patients, there were substantial rates of disagreement, ranging from 23.5% (nausea/vomiting) to 59.8% (fatigue) at 4 weeks. Of note, the disagreement was considerably higher when symptoms were reported by patients alone than by physicians alone (i.e., physicians’ underreporting), and the frequency of physicians’ underestimation ranged from 12.5% (nausea/vomiting) to 56.7% (fatigue). These findings highlight the importance of PROs as subjective measures of the incidence of symptomatic AEs to establish patient‐centered care in clinical practice. Therefore, PROs could bridge the considerable gap between the perceptions of patients and physicians regarding toxicity during treatments.

There was more disagreement in the patient and physician reporting of symptomatic AEs. Among the symptomatic AEs, the extent of the agreement was extremely low for more subjective symptoms, such as dyspnea and fatigue, than it was for relatively quantifiable symptoms, such as diarrhea and nausea/vomiting. Indeed, fatigue was the symptom that was most frequently underestimated by physicians in this study, and it has been consistently found to be the symptomatic toxicity that is, the most commonly underreported and that most impairs QOL in patients treated with chemotherapy.^7^ A possible reason for the underestimation by physicians might be that patient and physician reporting of symptomatic AEs was compared using different assessment instruments: the EORTC QLQ C‐30 by patients and the NCI‐CTCAE by physicians. The NCI‐CTCAE is generally used to assess any abnormal clinical findings that are temporally associated with the use of medical treatment but not the cancer itself by physicians, and it has a limitation from the psychometric perspective in valuing the symptom.^5^, ^32^ The EORTC QLQ‐C30 can assess patients’ QOL, including the psychosocial and functional aspects of their experience, instead of only providing simple reports of symptoms.^16^ Thus, the NCI‐CTCAE and EORTC QLQ‐C30 were intended for different purposes, and the conceptual and methodological differences might result in different perspectives reported by patients and physicians, especially for more subjective symptoms, such as dyspnea and fatigue, than for relatively quantifiable symptoms, such as diarrhea and nausea/vomiting.^32^ Moreover, for the relatively quantifiable symptomatic AEs, the grading of toxicity using the NCI‐CTCAE is based on the increase in number of stools for diarrhea or number of emesis episodes for nausea/vomiting to ensure consistency and standardization in assessments of treatment‐related AEs.^5^ However, nausea/vomiting and diarrhea may have a considerable impact on well‐being for patients, and the EORTC QLQ‐C30 can capture the global status of patients’ subjective perspectives and physical health.^16^ In fact, the proportion of underreporting remained relevant even when the analysis was limited to patients who reported “quite a bit” or “very much” toxicity on the EORTC QLQ‐C30 questionnaire. Other possible reasons for the disagreement and underestimation may include physician factors, such as less attention paid to toxicities that (a) were unrelated to the treatment, (b) did not prompt additional treatment or medication, (c) were largely expected with the drugs, (d) were already present before treatment, or (e) were related to cancer itself.^9^ The results of this study indicate the value of the EORTC QLQ‐C30 as a complementary tool for use with physician‐reported outcomes obtained using the NCI‐CTCAE. No clinicopathological factor was identified that could predict the disagreement for individual symptomatic AEs, consistent with previous reports.^7^ Considering the frequent physician underreporting, physicians should be aware of the possible risks of underestimating symptoms in all of their patients, especially for more subjective symptomatic AEs.

Despite the disagreement of some specific symptoms, physicians identify important problems that can predict clinical outcomes.^33^ Moreover, disagreement on the ECOG PS has been reported as an increased risk of death in patients with advanced cancers, including CRC,^34^ and the prognostic value of the disagreement between patient‐ and physician‐reported outcomes on symptomatic AEs during chemotherapy has not yet been determined in mCRC. To the best of our knowledge, this is the first study to report different outcomes as an independent prognostic factor despite the disagreement status not having effects on the treatment efficacies (ORR, PFS, and TTF) of cetuximab plus chemotherapy. Clinical trials usually exclude patients with comorbidities, patients with a severe symptom burden, and elderly patients at the time of study entry, which limits trials to relatively asymptomatic populations. In fact, most patients in this study had a good performance status (82.4% for ECOG PS0). For patients with a relatively good general condition and less tumor burden, cetuximab plus chemotherapy as the first‐line treatment may be feasible and tolerable for patients despite the disagreement status. Of note, patients with low agreement had a more unfavorable PPS than those with high agreement, indicating that there were detrimental effects of the disagreement status in the later‐line settings. Unlike their condition at the first‐line treatment, most patients have generally worsened ECOG PS and tumor‐related symptoms at a later‐line treatment. Therefore, early responsiveness to patient symptoms and appropriate management will be more important for patients receiving a later‐line of chemotherapy to prevent serious AEs, ensure lasting and effective chemotherapy, and deplete available drugs, which will contribute to prolonged survival. Patients with low agreement may have delayed responsiveness due to frequent physician underestimation and subsequently unfavorable PPS and OS. Although information on PROs was not provided to the physicians because of the independent collection in this study, the integration of PROs into patient care may improve the prognostic outcomes and facilitate early detection of treatment failure and unrecognized problems, which will build a firm patient–physician relationship, as demonstrated by several studies.^35^, ^36^, ^37^, ^38^ Thus, patients with low agreement may benefit from the routine use of PROs with feedback.

The main limitation of the present study was that it was not determined whether differences in race, ethnicity, physical background, education, and the status of communication affected the perceptions of symptoms. Several studies have shown that the time to first onset of most AEs was within the first 4 weeks of treatment and assessed patient and physician reporting at 4‐week time point.^39^, ^40^, ^41^ The 4‐week time point was expected to not only elicit the influence on the disagreement status of AEs but also minimize the influence of early study termination due to the first radiological assessment. In this study, the levels of agreement based on Cohen's κ values were similar throughout the study period (Table S1). In addition, the disagreement status at each time point of the assessment showed similar clinicopathological features and survival outcomes (Table S4 and Figure S4). However, the optimal frequency and timing when assessing the disagreement status remains unclear and needs to be addressed. Although location of the primary tumor (left‐sided colon vs. right‐sided colon) have different treatment efficacy for cetuximab plus chemotherapy,^4^ the concept of “tumor sidedness” had not yet been established when this study started. Therefore, we did not collect data on tumor sidedness in this study. Moreover, as most previous studies were monocentric or included patients with various types of cancer and treatments, the main strengths of this study are that it included a homogeneous study population (patients with mCRC), the use of a specific treatment, rigorous data collection throughout the study period, and high completion rates of questionnaires in a multicenter prospective study on QOL. Future carefully designed studies taking into account these limitations are required.

In conclusion, there were substantial rates of disagreement between patient‐ and physician‐reported outcomes on symptomatic AEs, which were associated with unfavorable outcomes in mCRC patients treated with cetuximab plus chemotherapy. The routine monitoring of patients’ symptoms using PROs may be useful to facilitate not only earlier recognition and management of symptoms but also patient‐centered care in clinical practice.

## AUTHORS’ CONTRIBUTIONS

Conception and design: Kensei Yamaguchi, Satoshi Morita, and Akira Ooki; Financial support: Junichi Sakamoto; Administrative support: Junichi Sakamoto; Provision of study materials or patients: All authors; Data management: Junichi Sakamoto; Statistical Analysis: Akira Ooki and Satoshi Morita; Interpretation: Akira Ooki and Satoshi Morita; Manuscript writing: Akira Ooki; Final approval of the manuscript: All authors; Accountable for all aspects of the work: All authors.

## Supporting information

Fig S1

Fig S2

Fig S3

Fig S4

Fig S5

Fig S6

Fig S7

Table S1

Table S2

Table S3

Table S4

## Data Availability

All data analyzed during this study are included in this published article and its supplementary information files.
