# Supplementary material for: Disagreement between patient‐ and physician‐reported outcomes on symptomatic adverse events as poor prognosis in patients treated with first‐line cetuximab plus chemotherapy for unresectable metastatic colorectal cancer: Results of Phase II QUACK trial
Source: Cancer Med. 2020 Nov 21;9(24):9419–30. doi: 10.1002/cam4.3564 (PMC7774728; doi:10.1002/cam4.3564)
Supplement: Supplementary file 1 — Fig S1 [file CAM4-9-9419-s001.pptx]

## Slide 1
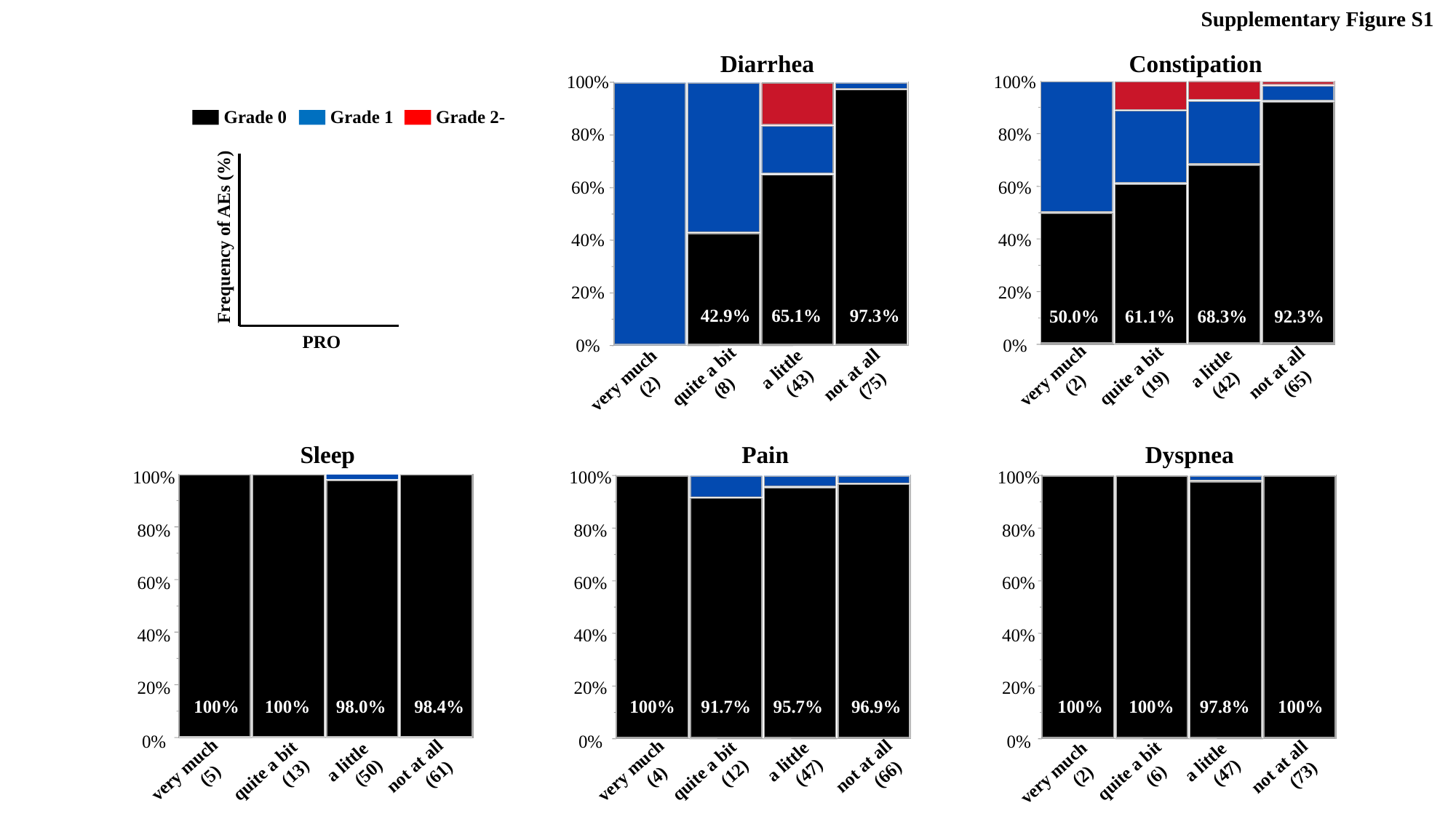

Supplementary Figure S1
Diarrhea
Constipation
100%
80%
60%
40%
20%
0%
100%
80%
60%
40%
20%
0%
Grade 0
Grade 1
Grade 2-
Frequency of AEs (%)
PRO
42.9%
 65.1%
 97.3%
50.0%
61.1%
 68.3%
 92.3%
a little
 (42)
not at all
 (65)
quite a bit
 (19)
very much
 (2)
a little
 (43)
not at all
 (75)
quite a bit
 (8)
very much
 (2)
Sleep
Pain
 Dyspnea
100%
80%
60%
40%
20%
0%
100%
80%
60%
40%
20%
0%
100%
80%
60%
40%
20%
0%
 100%
 100%
 98.0%
 98.4%
 100%
91.7%
 95.7%
 96.9%
 100%
100%
 97.8%
 100%
a little
 (47)
not at all
 (66)
quite a bit
 (12)
very much
 (4)
a little
 (47)
not at all
 (73)
quite a bit
 (6)
very much
 (2)
a little
 (50)
not at all
 (61)
very much
 (5)
quite a bit
 (13)
