# Supplementary material for: Disagreement between patient‐ and physician‐reported outcomes on symptomatic adverse events as poor prognosis in patients treated with first‐line cetuximab plus chemotherapy for unresectable metastatic colorectal cancer: Results of Phase II QUACK trial
Source: Cancer Med. 2020 Nov 21;9(24):9419–30. doi: 10.1002/cam4.3564 (PMC7774728; doi:10.1002/cam4.3564)
Supplement: Supplementary file 2 — Fig S2 [file CAM4-9-9419-s002.pptx]

## Slide 1
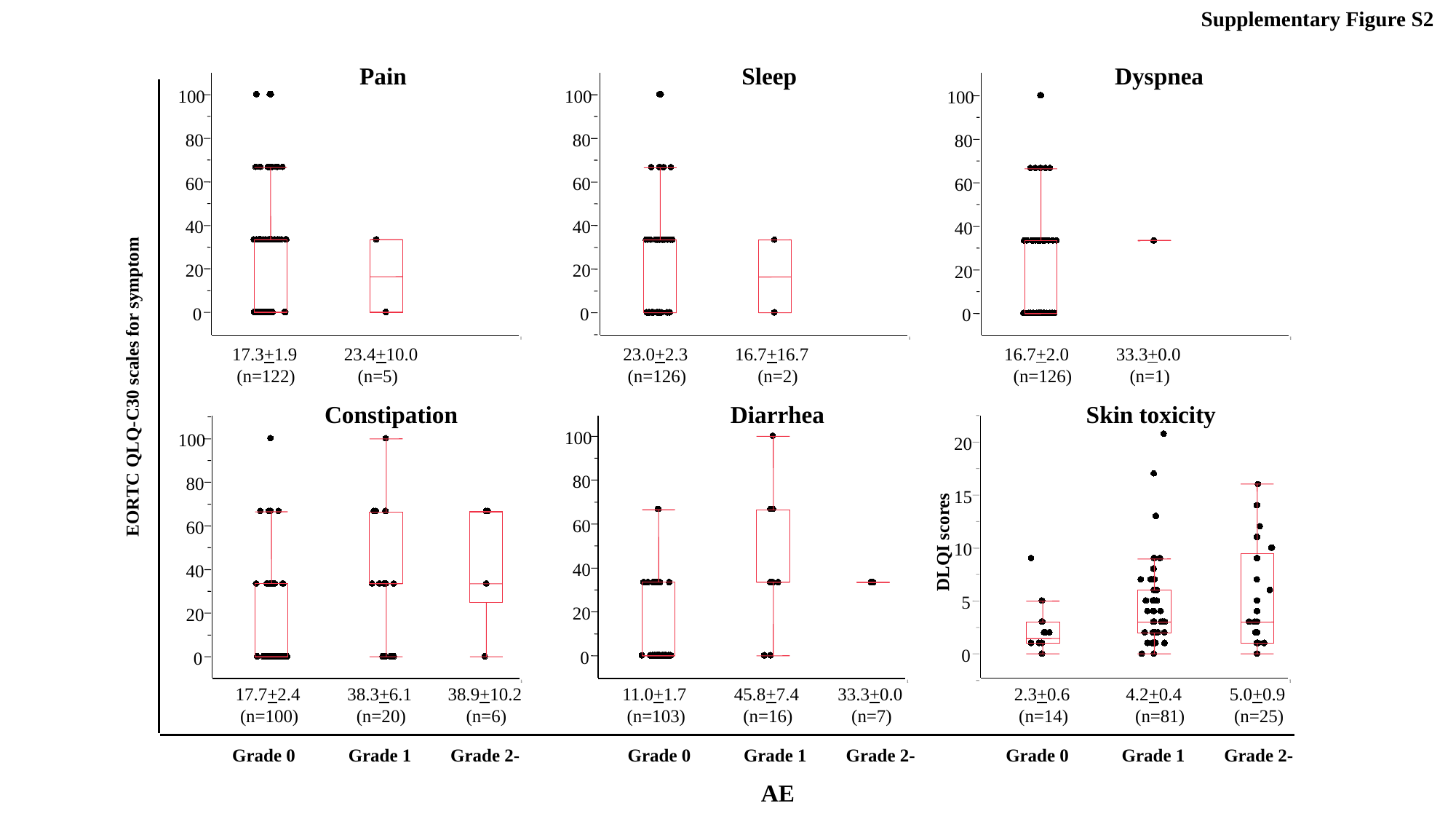

Supplementary Figure S2
Dyspnea
Pain
Sleep
100
80
60
40
20
0
100
80
60
40
20
0
100
80
60
40
20
0
16.7+2.0
 (n=126)
33.3+0.0
 (n=1)
17.3+1.9
 (n=122)
23.4+10.0
 (n=5)
23.0+2.3
 (n=126)
16.7+16.7
 (n=2)
 EORTC QLQ-C30 scales for symptom
Skin toxicity
Diarrhea
100
80
60
40
20
0
11.0+1.7
 (n=103)
45.8+7.4
 (n=16)
33.3+0.0
 (n=7)
Constipation
100
80
60
40
20
0
17.7+2.4
 (n=100)
38.3+6.1
 (n=20)
38.9+10.2
 (n=6)
20
15
10
5
0
 DLQI scores
2.3+0.6
 (n=14)
4.2+0.4
 (n=81)
5.0+0.9
 (n=25)
Grade 0
Grade 1
Grade 2-
Grade 0
Grade 1
Grade 2-
Grade 0
Grade 1
Grade 2-
AE
