# Supplementary material for: Disagreement between patient‐ and physician‐reported outcomes on symptomatic adverse events as poor prognosis in patients treated with first‐line cetuximab plus chemotherapy for unresectable metastatic colorectal cancer: Results of Phase II QUACK trial
Source: Cancer Med. 2020 Nov 21;9(24):9419–30. doi: 10.1002/cam4.3564 (PMC7774728; doi:10.1002/cam4.3564)
Supplement: Supplementary file 3 — Fig S3 [file CAM4-9-9419-s003.pptx]

## Slide 1
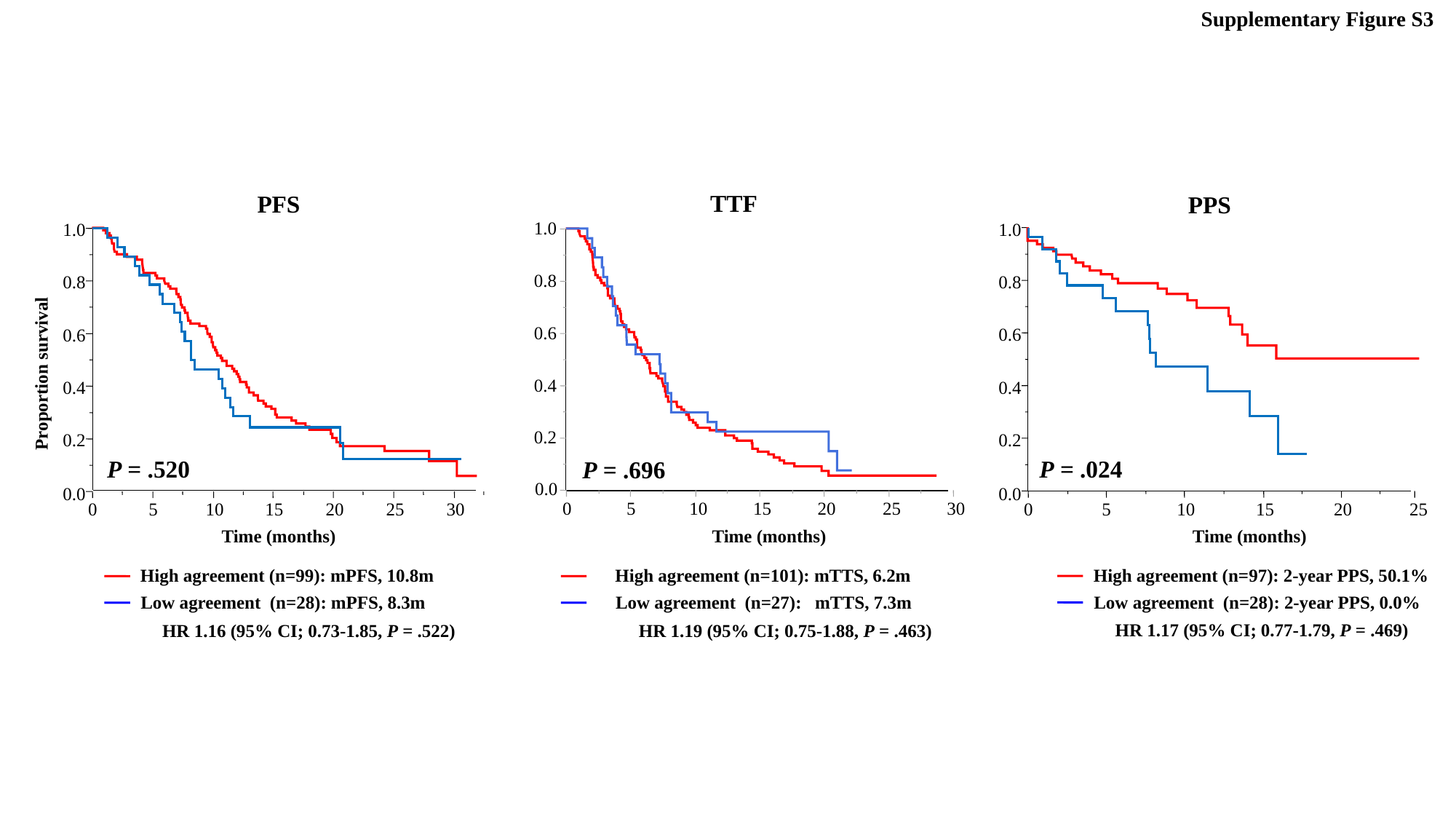

Supplementary Figure S3
 TTF
 PFS
 PPS
1.0
0.8
0.6
0.4
0.2
0.0
0
5
10
15
20
25
30
1.0
0.8
0.6
0.4
0.2
0.0
0
5
10
15
20
25
1.0
0.8
0.6
0.4
0.2
0.0
0
5
10
15
20
25
30
 Proportion survival
P = .520
P = .024
P = .696
Time (months)
Time (months)
Time (months)
High agreement (n=97): 2-year PPS, 50.1%
Low agreement (n=28): 2-year PPS, 0.0%
 HR 1.17 (95% CI; 0.77-1.79, P = .469)
High agreement (n=99): mPFS, 10.8m
Low agreement (n=28): mPFS, 8.3m
 HR 1.16 (95% CI; 0.73-1.85, P = .522)
High agreement (n=101): mTTS, 6.2m
Low agreement (n=27): mTTS, 7.3m
 HR 1.19 (95% CI; 0.75-1.88, P = .463)
