# Supplementary material for: Disagreement between patient‐ and physician‐reported outcomes on symptomatic adverse events as poor prognosis in patients treated with first‐line cetuximab plus chemotherapy for unresectable metastatic colorectal cancer: Results of Phase II QUACK trial
Source: Cancer Med. 2020 Nov 21;9(24):9419–30. doi: 10.1002/cam4.3564 (PMC7774728; doi:10.1002/cam4.3564)
Supplement: Supplementary file 4 — Fig S4 [file CAM4-9-9419-s004.pptx]

## Slide 1
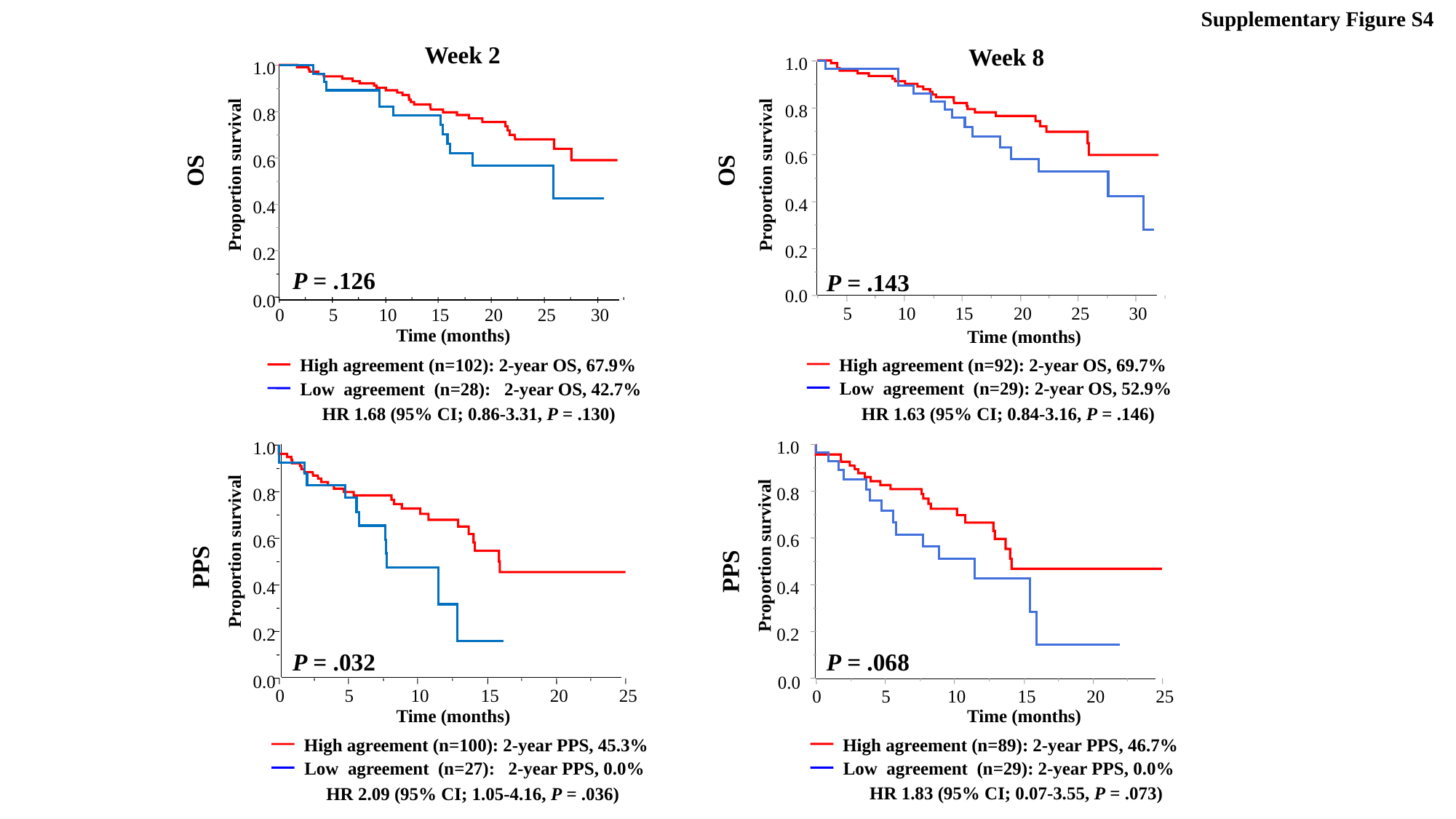

Supplementary Figure S4
 Week 2
 Week 8
1.0
0.8
0.6
0.4
0.2
0.0
5
10
15
20
25
30
1.0
0.8
0.6
0.4
0.2
0.0
0
5
10
15
20
25
30
 Proportion survival
 Proportion survival
 OS
 OS
P = .126
P = .143
Time (months)
Time (months)
High agreement (n=92): 2-year OS, 69.7%
Low agreement (n=29): 2-year OS, 52.9%
 HR 1.63 (95% CI; 0.84-3.16, P = .146)
High agreement (n=102): 2-year OS, 67.9%
Low agreement (n=28): 2-year OS, 42.7%
 HR 1.68 (95% CI; 0.86-3.31, P = .130)
1.0
0.8
0.6
0.4
0.2
0.0
0
5
10
15
20
25
1.0
0.8
0.6
0.4
0.2
0.0
0
5
10
15
20
25
P = .032
Time (months)
High agreement (n=100): 2-year PPS, 45.3%
Low agreement (n=27): 2-year PPS, 0.0%
 HR 2.09 (95% CI; 1.05-4.16, P = .036)
 Proportion survival
 Proportion survival
 PPS
 PPS
P = .068
Time (months)
High agreement (n=89): 2-year PPS, 46.7%
Low agreement (n=29): 2-year PPS, 0.0%
 HR 1.83 (95% CI; 0.07-3.55, P = .073)
