# Supplementary material for: Disagreement between patient‐ and physician‐reported outcomes on symptomatic adverse events as poor prognosis in patients treated with first‐line cetuximab plus chemotherapy for unresectable metastatic colorectal cancer: Results of Phase II QUACK trial
Source: Cancer Med. 2020 Nov 21;9(24):9419–30. doi: 10.1002/cam4.3564 (PMC7774728; doi:10.1002/cam4.3564)
Supplement: Supplementary file 5 — Fig S5 [file CAM4-9-9419-s005.pptx]

## Slide 1
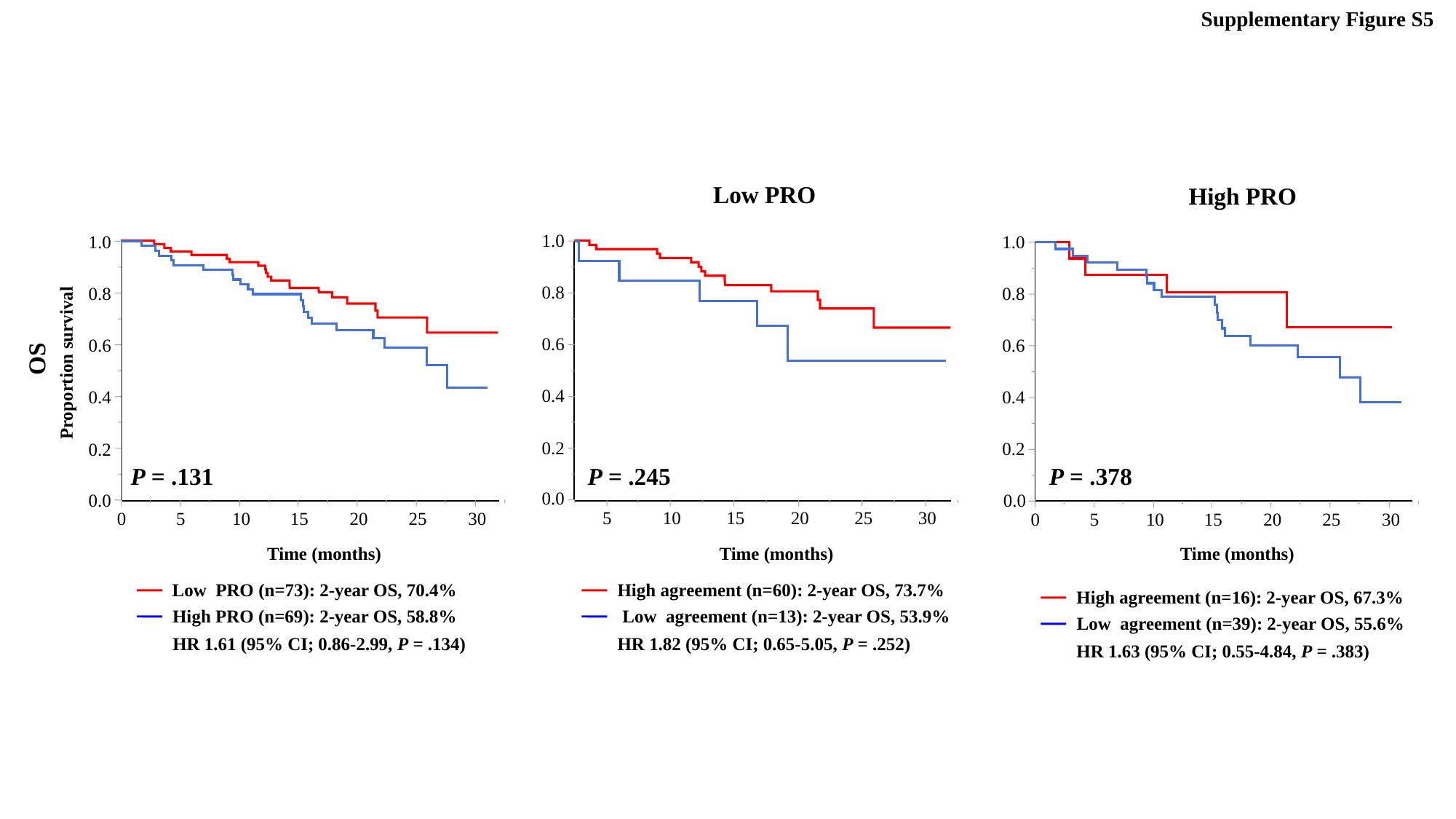

Supplementary Figure S5
 Proportion survival
 OS
1.0
0.8
0.6
0.4
0.2
0.0
0
5
10
15
20
25
30
P = .131
Time (months)
Low PRO (n=73): 2-year OS, 70.4%
High PRO (n=69): 2-year OS, 58.8%
 HR 1.61 (95% CI; 0.86-2.99, P = .134)
Low PRO
High PRO
1.0
0.8
0.6
0.4
0.2
0.0
5
10
15
20
25
30
P = .245
Time (months)
High agreement (n=60): 2-year OS, 73.7%
Low agreement (n=13): 2-year OS, 53.9%
 HR 1.82 (95% CI; 0.65-5.05, P = .252)
1.0
0.8
0.6
0.4
0.2
P = .378
0.0
0
5
10
15
20
25
30
Time (months)
High agreement (n=16): 2-year OS, 67.3%
Low agreement (n=39): 2-year OS, 55.6%
 HR 1.63 (95% CI; 0.55-4.84, P = .383)
