# Supplementary material for: Disagreement between patient‐ and physician‐reported outcomes on symptomatic adverse events as poor prognosis in patients treated with first‐line cetuximab plus chemotherapy for unresectable metastatic colorectal cancer: Results of Phase II QUACK trial
Source: Cancer Med. 2020 Nov 21;9(24):9419–30. doi: 10.1002/cam4.3564 (PMC7774728; doi:10.1002/cam4.3564)
Supplement: Supplementary file 6 — Fig S6 [file CAM4-9-9419-s006.pptx]

## Slide 1
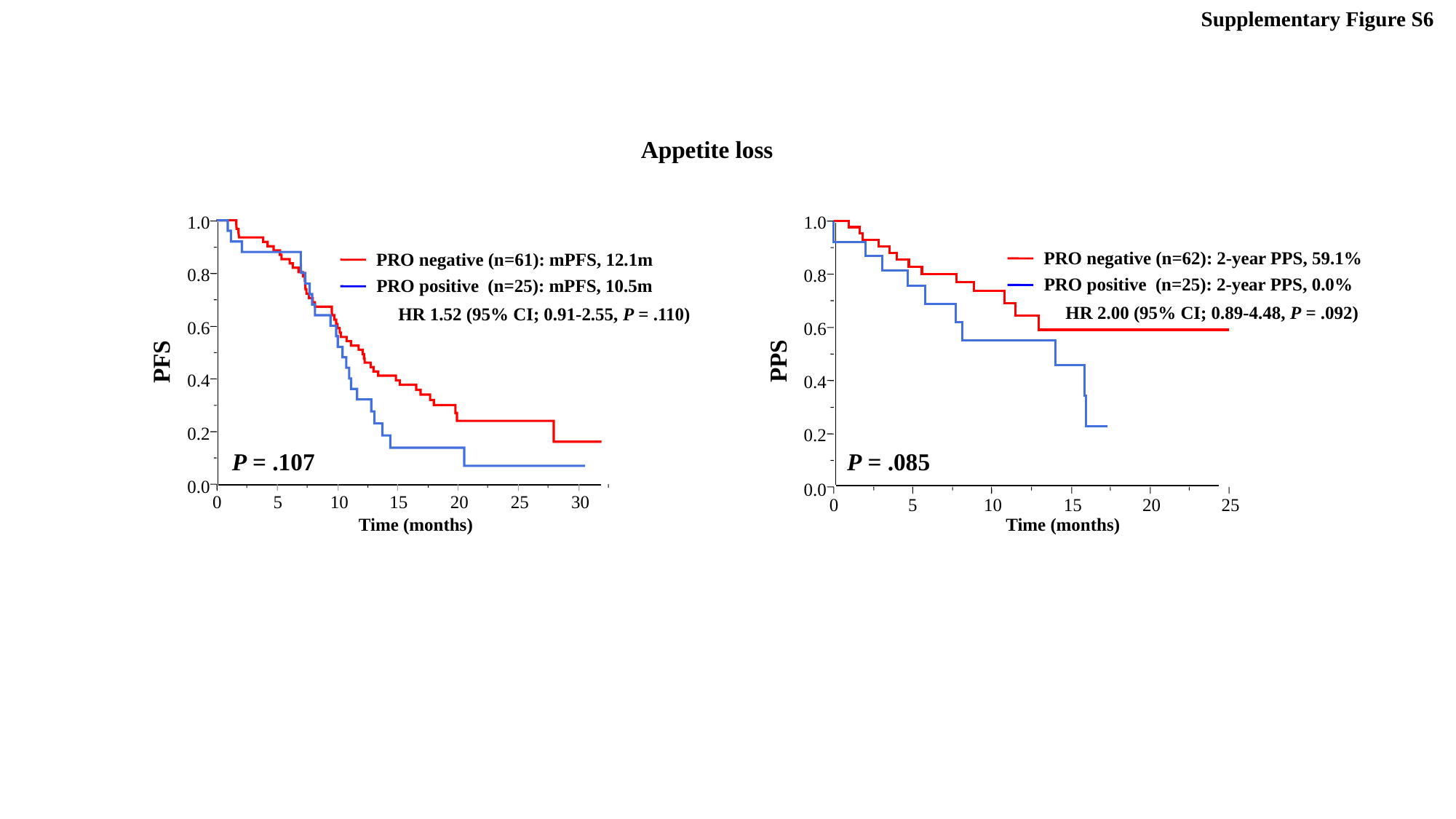

Supplementary Figure S6
Appetite loss
1.0
0.8
0.6
0.4
0.2
0.0
0
5
10
15
20
25
30
P = .107
Time (months)
1.0
0.8
0.6
0.4
0.2
0.0
0
5
10
15
20
25
PRO negative (n=62): 2-year PPS, 59.1%
PRO positive (n=25): 2-year PPS, 0.0%
 HR 2.00 (95% CI; 0.89-4.48, P = .092)
PRO negative (n=61): mPFS, 12.1m
PRO positive (n=25): mPFS, 10.5m
 HR 1.52 (95% CI; 0.91-2.55, P = .110)
 PFS
 PPS
P = .085
Time (months)
