# Supplementary material for: Disagreement between patient‐ and physician‐reported outcomes on symptomatic adverse events as poor prognosis in patients treated with first‐line cetuximab plus chemotherapy for unresectable metastatic colorectal cancer: Results of Phase II QUACK trial
Source: Cancer Med. 2020 Nov 21;9(24):9419–30. doi: 10.1002/cam4.3564 (PMC7774728; doi:10.1002/cam4.3564)
Supplement: Supplementary file 7 — Fig S7 [file CAM4-9-9419-s007.pptx]

## Slide 1
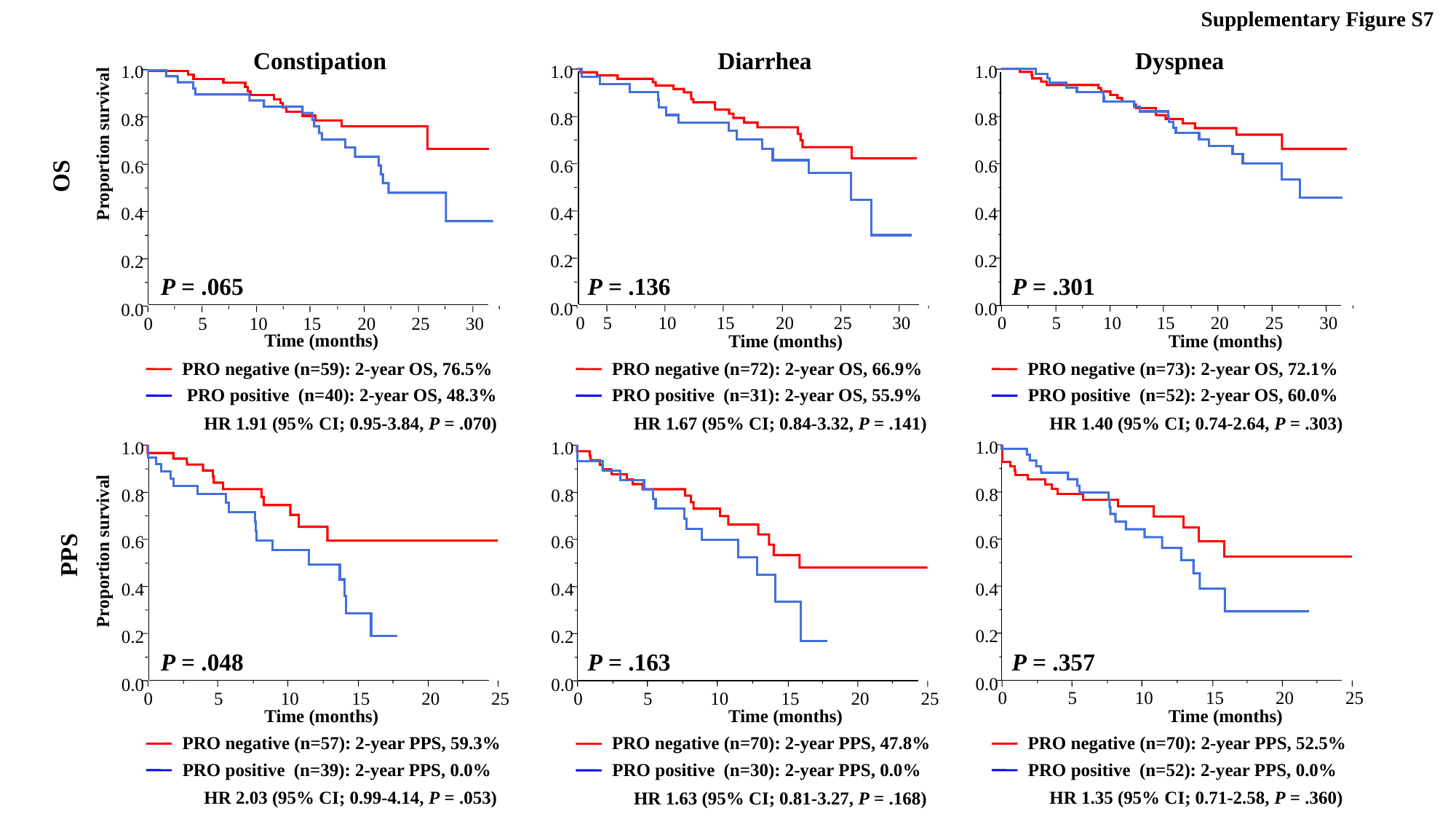

Supplementary Figure S7
Constipation
 Diarrhea
 Dyspnea
1.0
0.8
0.6
0.4
0.2
0.0
0
5
10
15
20
25
30
1.0
0.8
0.6
0.4
0.2
0.0
5
10
15
20
25
30
0
1.0
0.8
0.6
0.4
0.2
0.0
0
5
10
15
20
25
30
 Proportion survival
 OS
P = .065
P = .136
P = .301
Time (months)
Time (months)
Time (months)
PRO negative (n=72): 2-year OS, 66.9%
PRO positive (n=31): 2-year OS, 55.9%
 HR 1.67 (95% CI; 0.84-3.32, P = .141)
PRO negative (n=59): 2-year OS, 76.5%
PRO positive (n=40): 2-year OS, 48.3%
 HR 1.91 (95% CI; 0.95-3.84, P = .070)
PRO negative (n=73): 2-year OS, 72.1%
PRO positive (n=52): 2-year OS, 60.0%
 HR 1.40 (95% CI; 0.74-2.64, P = .303)
1.0
0.8
0.6
0.4
0.2
0.0
0
5
10
15
20
25
1.0
0.8
0.6
0.4
0.2
0.0
0
5
10
15
20
25
1.0
0.8
0.6
0.4
0.2
0.0
0
5
10
15
20
25
 Proportion survival
 PPS
P = .048
P = .163
P = .357
Time (months)
Time (months)
Time (months)
PRO negative (n=57): 2-year PPS, 59.3%
PRO positive (n=39): 2-year PPS, 0.0%
 HR 2.03 (95% CI; 0.99-4.14, P = .053)
PRO negative (n=70): 2-year PPS, 52.5%
PRO positive (n=52): 2-year PPS, 0.0%
 HR 1.35 (95% CI; 0.71-2.58, P = .360)
PRO negative (n=70): 2-year PPS, 47.8%
PRO positive (n=30): 2-year PPS, 0.0%
 HR 1.63 (95% CI; 0.81-3.27, P = .168)
